# Management and Microbiological Characteristics of Membrane Formation on a Hydrophilic Acrylic Intraocular Lens: A Clinical Case Series and Material Comparative Study of Different IOLs

**DOI:** 10.1155/2019/5746186

**Published:** 2019-02-03

**Authors:** Xiaodi Qiu, Yang Wu, Yongxiang Jiang, Yinghong Ji, Xiangjia Zhu, Jin Yang, Yi Lu

**Affiliations:** ^1^Eye Institute, Eye, Ear, Nose, and Throat Hospital of Fudan University, 83 Fenyang Road, Shanghai 200031, China; ^2^Key Laboratory of Myopia, Ministry of Health, Shanghai 200031, China; ^3^Shanghai Key Laboratory of Visual Impairment and Restoration, Shanghai 200031, China; ^4^Key Laboratory of Medical Molecular Virology of Ministries of Education and Health, Department of Medical Microbiology and Parasitology, School of Basic Medical Sciences, Fudan University, Shanghai, China

## Abstract

*Background*/*Aims*. To report a case series of membrane formation on intraocular lenses (IOLs) after uneventful phacoemulsification and to evaluate the material characteristics and biofilm formation on different IOLs. *Methods*. Ten eyes implanted with the same type of IOLs were found to have membranes on their IOLs after uneventful phacoemulsification from May 2015 to May 2016. No other patients were found with the same phenomenon among 11236 patients who underwent cataract surgeries during this period. To further investigate the reasons for their formation, we assessed seven types of IOLs used in our hospital, including their material characteristics and the presence of microbes (*Staphylococcus epidermidis*) on the IOL surface by scanning electron microscopy (SEM). All IOLs were incubated under *in vitro* flow conditions (BioFlux 1000Z). After 36 h, the IOLs were taken from the system, and both the bound bacteria and biofilm formation were observed. *Results*. Five eyes underwent intravitreal injections of ceftazidime and norvancomycin with one positive culture obtained from the anterior chamber fluid. The other five eyes only received topical treatment of gatifloxacin/levofloxacin and tobramycin. At the last follow-up, all patients had best-corrected visual acuity (BCVA) of 20/50 or better. In the biofilm study on the IOL surface, *Staphylococcus epidermidis* biofilms formed more readily on hydrophilic acrylic IOLs than on hydrophobic acrylic IOLs. *Conclusions*. Bacterial adhesion and biofilm tend to develop on certain types of IOLs because of the characteristics of the biomaterial.

## 1. Introduction

Postoperative endophthalmitis is a potentially sight-threatening infection which represents a therapeutic emergency [1, 2]. The mechanism of postoperative endophthalmitis has not been entirely elucidated to date. However, the binding of bacteria to intraocular lenses (IOLs) and biofilm formation are well known to be an essential step in the pathogenesis of endophthalmitis [3, 4]. Study of the biofilm formation appears to be essential for gaining a better understanding of the relationship between endophthalmitis [5] and the materials of the IOL [6–8]and developing new therapeutic or prevention strategies against endophthalmitis [9, 10].

Ten patients were consecutively found to have membrane formation on the same type of IOLs after uneventful IOL implantation in our hospital within one year. However, no other patients were found with a similar phenomenon using other types of IOLs during this time. This series of cases attracted our attention, and we wondered whether the biomaterial of this IOL may be more susceptible to bacterial adherence. There have been several previous studies focused on the bacterial adhesion to IOLs and biofilm formation and demonstrated that bacterial adhesion and biofilm development on the IOL surface depended on the characteristics of the biomaterial [5–8, 10, 11]. However, there were some discrepancies among these studies.

Here, we reported this case series of patients with membrane formation on the IOLs and evaluated their treatments and outcomes. The aim of this study was to develop an *in vitro* model to study *Staphylococcus epidermidis* biofilm formation and to compare the ability of *S. epidermidis* to form biofilms on various IOL materials as a step towards deepening the understanding of the interactions between pathogenic bacteria and IOL materials.

## 2. Patients and Methods

### 2.1. Demographics and Medical Histories

The Institutional Review Board (IRB) of the Eye and ENT Hospital of Fudan University approved this study, which adhered to the tenets of the Declaration of Helsinki. Informed consent was obtained from the subjects. The study included ten eyes of ten patients who were found to have membranes on their IOLs (QUATRIX Aspheric IOLs, Croma GmbH) for the treatment of age-related cataract from May 2015 to May 2016. Their medical records were reviewed (Table 1).

In our study, the detailed numbers of seven different types of IOLs implanted in cataract patients from May 2015 to May 2016 were also reported in Table 2. Patients with uveitis, glaucoma, or diabetic retinopathy (51 eyes) were implanted with the heparin surface-modified (HSM) hydrophilic acrylic IOLs.

### 2.2. Surgical Technique

All surgery was performed by two experienced doctors, using conventional phacoemulsification with IOLs implanted in the capsular bag. The postoperative medications given were levofloxacin and prednisolone acetate, four times/day for 2 weeks, and pranoprofen, four times/day for 4 weeks.

### 2.3. Intraocular Lenses

The study involved seven types of commercially available IOLs made from two different biomaterials: hydrophilic and hydrophobic acrylic (Table 2).

### 2.4. Bacterial Strain and Medium Composition

The Institute of Medical Microbiology of Shanghai Medical College of Fudan University provided the *S. epidermidis* strain 1457 (SE1457) described in the previous study [12]. B-medium and Tryptic soy broth (TSB, Oxoid, Cambridge, UK) were used for *S. epidermidis* cultivation and biofilm formation. The artificial aqueous humour was prepared by adding casein peptone (1.0 g/L), yeast extract (0.5 g/L), and glucose (1.0 g/L) in sterile physiological balanced salt solution.

### 2.5. Dynamic Biofilm Assay

Bacterial biofilms formation *in vitro* was performed using the BioFlux 1000Z microfluidic system (Fluxion Biosciences, Inc., San Francisco, CA, USA). The inlet wells of a BioFlux Plate (48 wells, 20 dyne) were seeded with a 10^8^ CFU/mL SE1457 bacterial solution by pumping from the inlet wells to the outlet wells at 2.0 dyne/cm^2^ for 8 s. The plate was incubated at 37°C for 30 min without flow to let bacteria cells adhere to the surface of the channels between the inlet and outlet wells. After cleaning the outlet wells, the IOLs were placed horizontally at the bottom. A total of 2.0 mL of artificial aqueous humour was added to the inlet wells and pumped at 37°C with a consistent flow (0.15 dyne/cm^2^) towards outlet wells for 36 h.

### 2.6. Scanning Electron Microscopy (SEM) Observations of IOLs and Biofilms

IOLs were fixed in 2.5% glutaraldehyde solution in a 0.1 M phosphate buffer for 2 h, followed by a secondary fixation with 1% osmium tetroxide stationary solution. Fixed IOLs were dehydrated in ethanol-water mixtures with increasing concentrations of ethanol and ethyl acetate. The IOL surface observations were performed at 10 kV with an SEM (JEOL JSM-6380LV; JEOL Ltd, Tokyo, Japan, and FEI Nova NanoSEM 450; FEI Ltd, Oregon, USA).

### 2.7. Quantification of Bacterial Adhesion

Gentle scraping of both optic surfaces of IOLs was performed to remove the adhered bacteria cells. Bacterial aggregates were subsequently dissociated through the needle of a syringe and vortexed for 3 min. The resultant suspension was then diluted and spread over a nutritive agar plate (TSB). Colonies were counted as colony-forming units (CFU)/mm^2^ after 24 h of incubation at 37°C.

### 2.8. Data Analysis

Snellen VAs were converted to the logarithm of the minimum angle of resolution (logMAR) values for data analyses. All statistical analyses were performed using SPSS for Windows (ver. 13.0, SPSS, Inc., Chicago, IL, USA). Mean values with the standard deviation of the mean (±SD) are reported unless otherwise stated. *P* values <0.05 were considered statistically significant and of <0.01 highly significant.

## 3. Results

### 3.1. Clinical Case Series

#### 3.1.1. Treatments and Outcomes

From May 2015 to May 2016, there were 11236 patients who received implantations of IOLs in our hospital. Ten patients were found to have membrane formation on the same type of IOLs (Table 1), and no membrane formation was found on any other types of IOLs (Table 2). The incidence of endophthalmitis with membrane formation on the IOL surface was 0.89%. These patients had a mean age of 66.50 ± 8.63 years, and the median time until membrane development was found to be 27.50 ± 20.90 days.

Among these patients, five of ten eyes received long-term tropical treatment of gatifloxacin/levofloxacin and tobramycin. Three of ten eyes underwent intravitreal injections of norvancomycin 1 mg/0.1 mL and ceftazidime 2.25 mg/0.1 mL, and two of ten eyes underwent pars plana vitrectomy (PPV) plus intravitreal injections of ceftazidime and norvancomycin. All of the patients were treated with long-term (at least one month) tropical treatment of gatifloxacin/levofloxacin and tobramycin. At the last follow-up, all patients had best-corrected visual acuity (BCVA) of 20/50 or better. The ten eyes had a mean preoperative logMAR VA of 0.800 ± 0.267, which decreased after membrane formation (logMAR VA of 1.160 ± 0.350). Their final logMAR BCVA improved to 0.190 ± 0.120 at the last visit after treatment compared with VA before cataract surgery (*P* < 0.001).

There were three types of patients: acute onset, chronic onset, and recurrent membrane formation. We report the representative cases below.

#### 3.1.2. Acute Onset Membrane Formation (Case 7)

**Figure 1:**
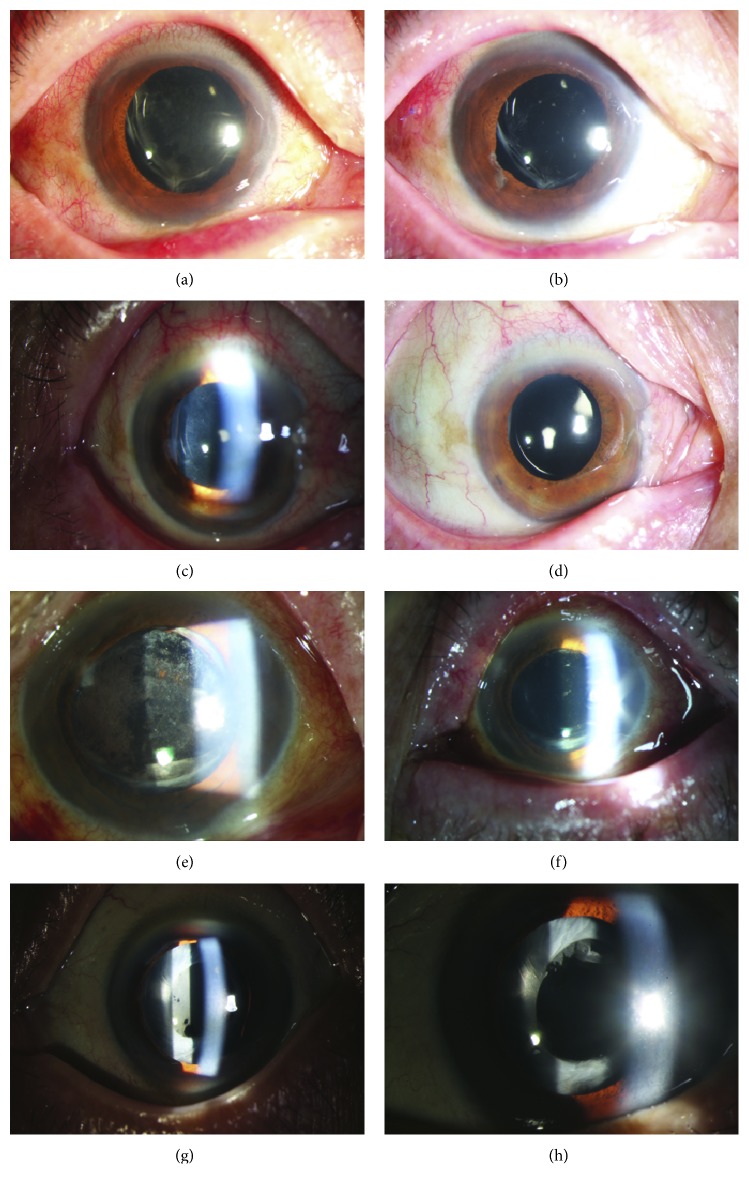
Slit-lamp images of eyes before and after treatment. (a) Case 4 before treatment. (b) Case 4 after treatment. (c) Case 7 before treatment. (d) Case 7 after treatment. (e) Case 8 before treatment. (f) Case 8 after treatment. (g) Case 9 before treatment. (h) Case 9 after treatment.

A 67-year-old female complained of visual loss, pain, and a foreign body sensation in the operated eye three days after surgery. On examination, VA of the right eye was 20/200 and found membrane formation around the IOL and capsule with 2+ anterior chamber cell and flare (Figure 1). Dilated fundus examination showed mild to moderate vitreous opacification. This patient was then treated with a vitreous injection of intravitreal ceftazidime and norvancomycin, as well as topical medication. The anterior chamber and vitreous culture were negative. After one week, her VA improved to 20/50.

#### 3.1.3. Chronic Onset Membrane Formation (Case 8)

A 55-year-old man complained of a two-month history of visual blurriness and photophobia (Figure 1). The symptoms appeared 3 weeks after the surgery. On examination, visual acuity of the right eye was 20/2000 and IOP was 32.6 mmHg. Dilated fundus examination showed moderate vitreous opacification. This patient was treated with PPV and vitreous injection. Inflammatory exudate from the anterior chamber culture was obtained by needle tap and subsequently grew *S. epidermidis* sensitive to levofloxacin and resistant to clindamycin. Postoperatively, this patient was treated with tropical medication. After one month, his VA improved to 20/100.

#### 3.1.4. Recurrent Membrane Formation (Case 6)

A 58-year-old female complained of visual loss and redness in the operated eye forty days after surgery. On examination, VA of the right eye was 20/200. Slit-lamp examination found membrane formation on the anterior optic part of the IOL with 1 + anterior chamber flare. Dilated fundus examination showed mild vitreous opacification. The patient was diagnosed with noninfectious iritis and anterior capsular opacification. After two weeks of topical application of corticosteroids until the inflammation subsided, this patient received Nd:YAG capsulotomy. However, the membrane on the IOL appeared again two days after capsulotomy with 4 + anterior chamber cell, hypopyon, and anterior chamber fibrin, with an IOP of 40 mmHg. B-scan demonstrated mild to moderate vitreous opacification. The patient then underwent PPV and intravitreal injections, with tropical medication. The patient demonstrated clinical improvement, and two weeks later, her visual acuity improved to 20/40, with an IOP of 14 mmHg.

### 3.2. Comparative Study of Different IOLs

In our study, we compared the characteristics of seven types of IOL used in our clinical practice (Table 2), including the material characteristics and the presence of *S. epidermidis* biofilms on the IOL surface by SEM.

#### 3.2.1. SEM of IOLs

**Figure 2:**
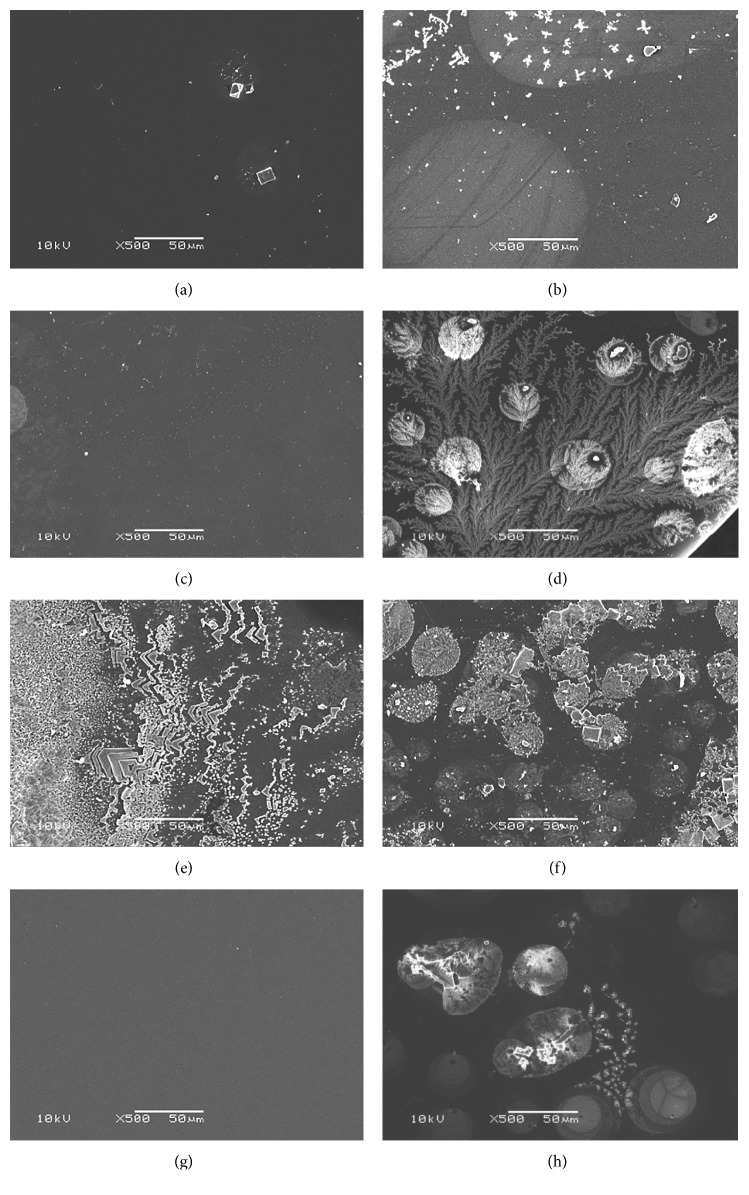
Scanning electron microscopy of different IOLs. (a) Human Optics MCX11ASP. (b) Hexa Vision HQ-201HEP. (c) Rayner 970C. (d) Croma QUATRIX Aspheric. (e) Croma QUATRIX Aspheric. (f) Croma QUATRIX Aspheric Evolutive. (g) Alcon SN60WF. (h) Abbott TECNIS ZA9003.

The hydrophobic acrylic IOLs showed a smooth surface or rare tiny fragments (Figure 2). However, the hydrophilic acrylic IOLs reflected varied manifestations. Two types of hydrophilic acrylic IOLs had tiny fragments on their surface (Figures 2(a) and 2(c)). Heparin surface-modified (HSM) hydrophilic acrylic IOLs were found to have dendritic crystals (Figure 2(b)). There were massive fern-like or shield-like crystals on two types of hydrophilic acrylic IOLs (Figures 2(d)–2(f)).

#### 3.2.2. SEM of Biofilm Development on IOLs

**Figure 3:**
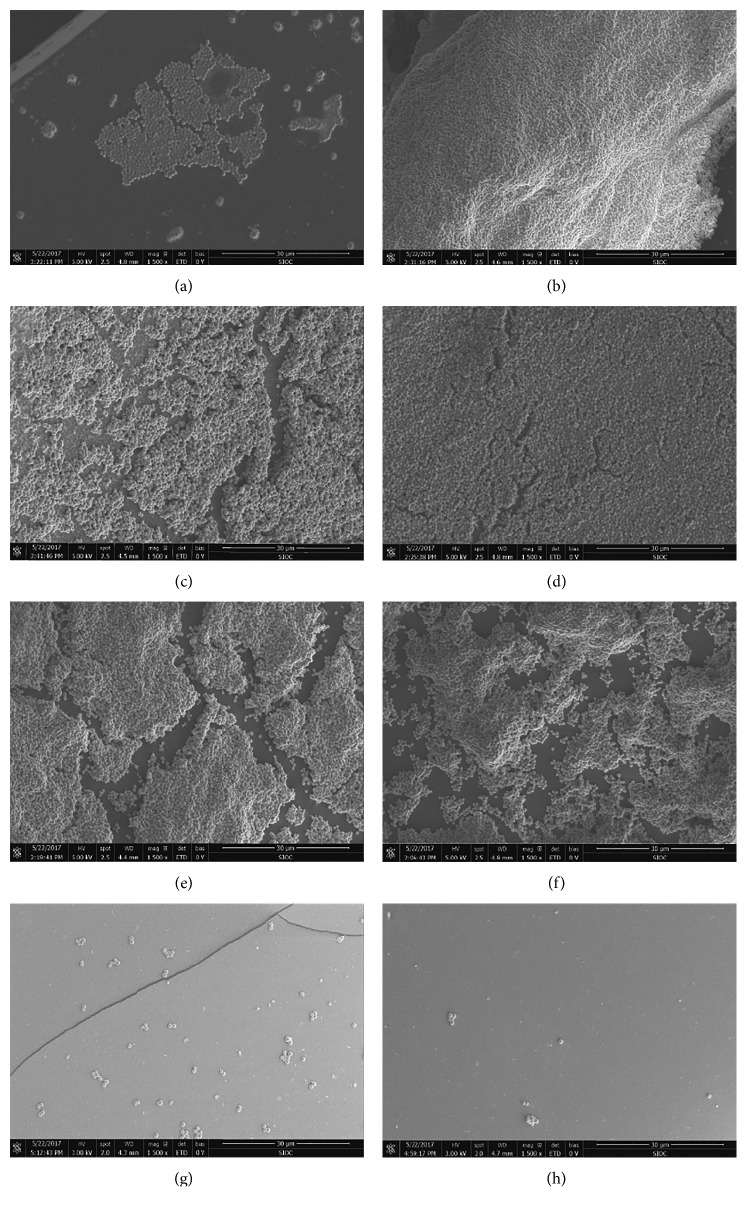
Scanning electron microscopy of bacterial biofilm on different IOLs. (a) Human Optics MCX11ASP. (b) Hexa Vision HQ-201HEP. (c) Rayner 970C. (d) Croma QUATRIX Aspheric. (e) Croma QUATRIX Aspheric. (f) Croma QUATRIX Aspheric Evolutive. (g) Alcon SN60WF. (h) Abbott TECNIS ZA9003.

There were isolated or aggregated cocci on all types of IOL optic surfaces by SEM. As depicted in Figure 3, SE1457 appeared to adhere more to hydrophilic IOLs than to hydrophobic IOLs, and it was found that the biofilm formation on the hydrophobic IOLs was the lowest among the materials tested (Figures 3(g) and 3(h)). As observed in Figure 3, there were no significant differences among the three brands of hydrophilic acrylic IOLs at 36 h (Figures 3(c)–3(f)). However, there were significant differences between brands of hydrophilic IOLs (Figures 3(a)–3(f)). The biofilms on HSM hydrophilic acrylic IOLs were significantly greater than those on the other materials after 36 h.

#### 3.2.3. Bacterial Population Enumeration in IOL Biofilms

After 36 h of incubation, the number of bound bacteria per unit area increased the most from hydrophobic acrylic to hydrophilic acrylic (Table 3). Table 3 shows the differences in bacterial adhesion to the biomaterials (*P* < 0.001). Quantitative plate counting revealed no significant difference in the attached bacterial numbers of adherent bacteria among the two hydrophobic IOLs. SE1457 appeared to adhere more to the hydrophilic lens than to the hydrophobic IOLs, but the difference was not statistically significant (*P* < 0.05 in F vs B, F vs E, G vs B, G vs D, and G vs E). As illustrated in Table 3, there were no significant differences in the number of adherent bacteria between the F brand and the G brand of hydrophobic IOLs (*P* > 0.05). However, significant differences were observed among the hydrophilic IOLs. Specifically, the A-brand hydrophilic acrylic IOLs were distinct from the four other hydrophilic acrylic IOLs (*P* < 0.05); HSM hydrophilic acrylic IOLs had the most bacteria, but there were no significant differences compared with D-brand and E-brand hydrophilic acrylic IOLs (*P* > 0.05).

## 4. Discussion

Postoperative bacterial endophthalmitis is considered to be one of the most feared complications and needs urgent treatment [13]. These ten patients in our study were all treated for endophthalmitis even though only one patient was found to have a positive culture. Our active anti-infection management proved effective and obtained a good visual outcome. Management of postoperative bacterial endophthalmitis has been explored in many studies [13]. Whether to perform lensectomy and posterior capsule stripping as a first-line procedure, along with vitreous tap and intravitreal injections of antibiotics, has been discussed in previous studies [2, 13, 14]. If the clinical phenomenon is not improving after intravitreal antibiotics within 24 h, or culture of a biofilm-producing organism is positive, vitrectomy should be considered urgently with vitreous cavity antibiotic administration. We recommend that lensectomy and posterior capsule stripping should be performed only in cases where the infection proves to be resistant to antibiotics.

Organism isolation is the fundamental basis of treatment. The initial cultures performed in our study were mostly negative. Prophylactic antibiotics may affect bacterial isolates [15]. Furthermore, previous reports have suggested that some bacteria will only grow on special culture media or under certain conditions [16]. In our case series, all cultures grew on the routine culture medium. The only positive culture found was *S. epidermidis*, which commonly exists on human skin and may be able to cause opportunistic infections.

Staphylococcal biofilm formation is modulated by many variables, including environmental factors such as bacterial strains, type of medium, temperature, hydrodynamic forces, different experimental protocols, and surface characteristics, which may be reasons for the different results among studies [17, 18]. We applied the BioFlux 2000 microfluidic system in order to replicate intraocular physiological conditions and hydrodynamics. Baillif's study showed that the stabilization phase was reached after 28 h of incubation [19]. In our study, we incubated the IOLs for 36 h to maximize the difference among groups.

Bacteria embedded in a biofilm are more able to resist attacks by antibiotics or host defences, and biofilm bacteria can survive the use of antiseptics and/or antibiotics at extremely high concentrations, which may lead to persisting infections [20, 21]. At that point, removing the infected device should be considered to end the infection. In our study, there was membrane formation in all patients during the routine anti-infection medication treatment, and it took quite a long time for the anti-infection treatment afterward to be effective.

In our study, bacterial adhesion and colonization were strongest both on the heparin surface-modified (HSM) hydrophilic acrylic IOL and the IOL used in our ten patients and weakest on the hydrophobic acrylic polymer. This may be related to the surface modification and IOL material. The heparin could inhibit inflammation for certain patients, but this modification may meanwhile increase the bacterial adherence. However, there were no cases of endophthalmitis among patients given HSM hydrophilic acrylics IOLs. This may be related to the rare application of this type of IOL in our clinical practice. Biofilm formation on polymer surfaces is a complex process that depends on the bacterial characteristics, the nature of the polymer material, and environmental factors. However, among all of the nonspecific interactions, hydrophilic/hydrophobic interactions had the greatest influence on the bacterial primary attachment [11]. Bacteria with hydrophilic properties generally prefer hydrophilic material surfaces [22–25]. Furthermore, different isolates of *S. epidermidis* may differ significantly with regard to their adherence to the same IOL [24, 25]. This may explain the discrepancy between our study and previous studies. Furthermore, a large number of crystals and a rougher surface than found on hydrophobic IOLs may contribute to more bacteria adhering to the surface of hydrophilic acrylic IOLs.

## 5. Conclusions

It is obviously difficult to draw definitive conclusions from our case series, and further clinical experience and research are necessary to validate our results. However, we hereby present a case series of endophthalmitis to highlight the importance of biofilm production and discuss the treatments. The findings in the present study of bacterial biofilm formation on the surface of IOLs allow for further understanding of biomedical device-related infections such as endophthalmitis. Furthermore, designing a material that could reduce or inhibit bacterial adhesion and growth on its surfaces to decrease the incidence of endophthalmitis should be a future research concern.

## Figures and Tables

**Table 1:** The demographics of the ten cases implanted with QUATRIX Aspheric IOLs.

Case no.	Age	Gender	Medical history	Ophthalmic history	Preoperative VA	Onset (days)	VA on POE	Anterior chamber	Vitreous opacification	AC/vitreous tapping	Tropical treatment	Systemic treatment	Final VA	Final BCVA
1	74	M	—	AMD	20/100	32	20/200	Tyn (+) Cell (+)	Mild	No	Levofloxacin and tobramycin eye drop	Oral levofloxacin	20/50	20/25
2	70	F	Hypertension	—	20/200	55	20/400	Tyn (++) Cell (++)	Mild to moderate	Yes, (-)	Intravitreal ceftazidime + norvancomycin	Intravenous levofloxacin	20/80	20/40
3	64	M	—	—	20/80	5	20/200	Tyn (+) Cell (+)	Mild	No	Gatifloxacin and tobramycin eye drop	Intravenous levofloxacin	20/40	20/25
4	74	F	—	—	20/100	9	20/100	Tyn (+) Cell (+)	Mild	No	Gatifloxacin and tobramycin eye drop	Oral levofloxacin	20/40	20/32
5	76	F	Hypertension	—	20/200	26	20/200	Tyn (++) Cell (++)	Mild to moderate	Yes, (-)	Intravitreal ceftazidime + norvancomycin	Intravenous levofloxacin	20/80	20/50
6	58	F	Diabetes	—	20/200	40	20/400	Tyn (+++) Cell (++++)	Mild to moderate	Yes, (-)	YAG + PPV + Intravitreal ceftazidime + norvancomycin	Oral levofloxacin	20/40	20/25
7	67	F	—	—	20/100	3	20/200	Tyn (+) Cell (+)	Mild to moderate	No	Gatifloxacin and tobramycin eye drop	Oral levofloxacin	20/50	20/32
8	56	M	—	Pathological myopia	20/400	65	20/2000	Tyn (+++) Cell (++)	Moderate	Yes, *S. epi*	PPV + Intravitreal ceftazidime + norvancomycin	Oral levofloxacin	20/100	20/40
9	74	F	—	—	20/80	24	20/200	Tyn (+) Cell (+)	Mild to moderate	No	Gatifloxacin eye drop + YAG	Oral levofloxacin	20/20	20/25
10	52	M	—	Pathological myopia	20/50	16	20/400	Tyn (++) Cell (++)	Mild to moderate	Yes, (-)	Intravitreal ceftazidime + norvancomycin	Oral cefaclor	20/50	20/32

AMD = age-related macular degeneration; VA = visual acuity; BCVA = best-corrected visual acuity; PPV = pars plana vitrectomy; YAG = yttrium aluminium garnet.

**Table 2:** Intraocular lens characteristics.

Group	Material	Manufacturer	Model number	Style of haptics	Eyes implanted with the IOL (May 2015–May 2016)
A	Hydrophilic acrylic	Human Optics AG	MCX11ASP	1-piece	998
B	Hydrophilic acrylic (heparin surface modification)	Hexa ision SARL	HQ-201HEP	1-piece	51
C	Hydrophilic acrylic	Rayner Intraocular Lenses Limited	970C	1-piece	2279
D	Hydrophilic acrylic	Croma GmbH	QUATRIX Aspheric	1-piece	461
E	Hydrophilic acrylic	Croma GmbH	QUATRIX Aspheric Evolutive	1-piece	NA
F	Hydrophobic acrylic	Alcon Laboratories, Inc.	SN60WF	1-piece	800
G	Hydrophobic acrylic	Abbott Medical Optics, Inc.	TECNIS ZA9003	3-piece	153

**Table 3:** Bacterial population enumeration in IOL biofilms (CFU/mm^2^).

Group	A	B	C	D	E	F	G
	6886	132631	58670	118483	76041	35368	37490
	8710	107873	45632	86652	90189	47747	31124
	10524	99031	40743	93726	68968	33600	33423
Mean ± SD	8706 ± 1819	113178 ± 17416	48348 ± 9267	99620 ± 16714	78399 ± 10805	38905 ± 7708	34012 ± 3223

## Data Availability

The clinical data used to support the findings of this study were provided by the Eye and ENT Hospital of Fudan University under license and so cannot be made freely available. Access to these data will be considered by the author upon request, with permission from the Eye and ENT Hospital of Fudan University. The laboratory research data used to support the findings of this study are included within the article.
